# Supplementary material for: The wisdom of stalemates: consensus and clustering as filtering mechanisms for improving collective accuracy
Source: Proc Biol Sci. 2020 Nov 4;287(1938):20201802. doi: 10.1098/rspb.2020.1802 (PMC7735266; doi:10.1098/rspb.2020.1802)
Supplement: Supplemantary Material [file rspb20201802supp1.pdf]

The wisdom of stalemates: consensus and clustering as filtering mechanisms for improving collective accuracy

Supplementary Material

S1 Time to consensus increases linearly with group size

Figure S1A shows the average time Watts-Strogatz networks with different parameters setting need to reach consensus. For group sizes up to 350 (i.e the range discussed in this project) we find that the linear function $f(n) = 6n$ is a practical upper bound. From these results we can be reasonably certain that a network that hasn't reached consensus within 10000 updating steps is stuck in a stalemate, which (apart from the breaking condition described in the main text) we use as a final breaking condition to avoid overly long simulations.

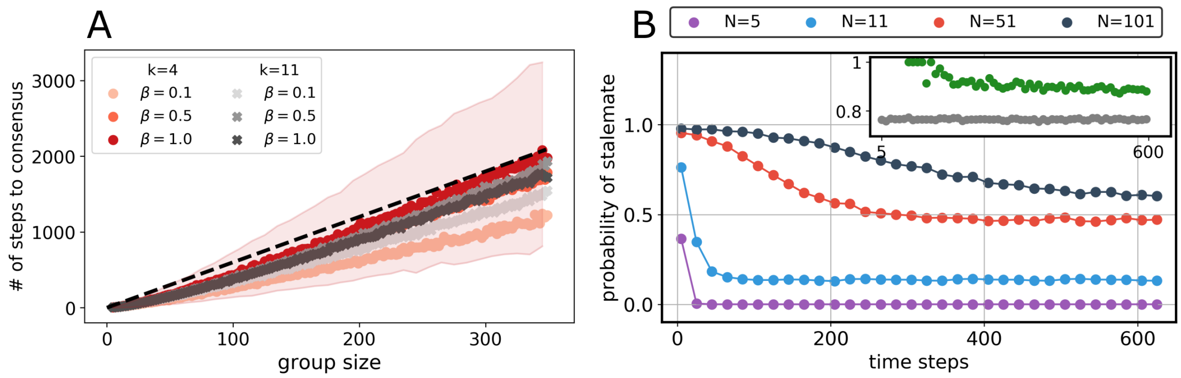

Figure S1. **A. Number of updating steps needed for consensus:** Comparison of saturation time for six different types of Watts-Strogatz networks. The number of nearest neighbors is varied from 'low' ($k = 4$ red lines) to 'high' ($k = 11$, grey lines), and the reconnection probability β can take low ($\beta = 0.1$, light colors), medium ($\beta = 0.5$) or high ($\beta = 1.0$, dark colors) values. Overall we find that saturation time increases approximately linearly with group size. For group sizes in the range investigated in this work (up to $N = 350$) the time to consensus is reasonably bounded by a a linear function of group size. The area shaded in light red, indicates the standard deviation in saturation times for the slowest networks ($k = 4, \beta = 1$). **B.** Probability of stalemates as a function of the maximum number of updating steps (s_{max}) for groups of different sizes ($N = 5$ to 101). Small groups will almost always reach consensus after as few as 50 opinion changes. Larger collectives generally require more interactions to reach consensus and therefore only groups which are highly biased initially will manage to do so when interaction time is limited. Stalemate filtering leads to a decrease in collective accuracy as interaction time increases. The inset shows the effect of s_{max} on collective accuracy for a group of $N = 51$ individuals in a simple environment with a cue of reliability $r = 0.55$. Green dots show collective accuracy at consensus and grey dots the result of a majority vote.

S2 Faster decision bouts lead to increased collective accuracy

If the number of number of individual opinion updates s_{max} is limited, networks are generally less likely to come to consensus. in agreement with our results about network structure we find that the harder consensus is to reach, the more likely it is to be accurate when reached (see Figure S1 B). The reasoning is similar to that for clustered and sparse networks: in order for groups (particularly large ones) to reach consensus rapidly (*i.e.*, when s_{max} is small), the set of initial opinions must be highly biased, which in the case of positively informative cues tends to occur more often for the correct option.

This result is seemingly in conflict with the well-known speed-accuracy trade-off, whereby higher decision accuracy typically comes with the cost of a greater amount of time needed to accumulate information and make a decision [1, 2]. In these cases, the trade-off is a direct consequence of the decision threshold, which determines the amount of evidence necessary for a decision to be made. If the threshold is high, decisions require large amounts of evidence, leading to longer integration time but

also robustness against noise and therefore higher accuracy. By contrast, lower thresholds require less evidence and allow faster decisions. These fast decisions are more susceptible, however, to random fluctuations and are therefore less accurate.

In our model, the speed-accuracy trade-off appears to be violated *within* a given decision bout, since in general, faster decisions (smaller s_{max}) lead to higher accuracy (Figure S1 B). However, this trade-off is restored when viewed *across* multiple decision bouts. When s_{max} is small, each decision bout is short, but the number of bouts needed before an initial condition is found that is sufficiently biased to reach consensus increases. From this perspective, the total amount of time needed before a consensus decision is made increases as s_{max} decreases, while the decision accuracy at consensus also increases thus restoring the speed-accuracy trade-off.

S3 Scale-Free networks are highly likely to reach consensus

While the Watts-Strogatz family already covers a wide range of network types from very clustered to completely random we also studied the effects of different graph types on the stalemate filtering. Most interestingly, we found that on scale-free networks such as the ones produced by the Barabasi-Albert model ([3]) the probability of consensus is almost 100% independent of group size (up to N=200, see Figure S2 B). Only when the average out-degree is chosen extremely low (e.g $m = 2$ in Figure S2 B) a significant proportion of decision trials does end in stalemates. This lack of stalemates undermines the benefits of stalemate filtering and the collective accuracy at consensus is similar to the collective accuracy achieved by majority voting. This is confirmed by the example in figure S2 C, where we see that collective accuracy at consensus is not much higher than the accuracy achieved by majority voting. In particular, we find that even for scale-free graphs with very low out-degree ($m = 2$) the accuracy boost due to stalemates is substantially lower than in a much more densely connected Watts-Strogatz graph.

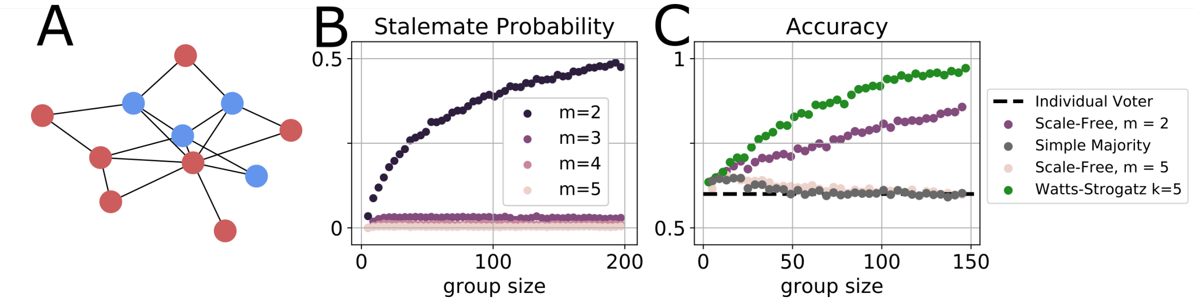

Figure S2. **Stalemates on a Scale-Free Network:** A) an example of a scale-free network generated with the Barabasi-Albert Model B) Probability of a decision trial ending in stalemate as a function of group size. Only for very low out-degree ($m = 2$, purple line) do networks get stuck in a stalemate. C) Because scale- free networks almost always reach consensus, the collective accuracy at consensus is similar to the collective accuracy achieved by majority voting (pink vs grey lines). If the out-degree is very low ($m = 2$, purple line), accuracy will increase with group size but less strongly than in a Watts-Strogatz graph with higher connectivtiy.

S4 Stalemate filtering in real fish networks suggests advantages of cohesion with respect to consensus frequency

To study the impact of stalemate filtering on realistic biological networks we applied our model to data from [4]. In these experiments schooling fish (golden shiners, *Notemigonus crysoleucas*) were exposed to Schreckstoff, a natural alarm substance, released from fish skin in the presence of a successful attack. A sensation of Schreckstoff will trigger a fear response increasing group cohesion [4]. To study the effect of network structure on stalemate probability and decision accuracy we used position data before and after the release of Schreckstoff and inferred the network structure by using a simple spatial network generation algorithm [5]. In particular, two individuals were considered neighbors in the network, if they were less than 16cm apart. We further only considered networks that were connected both before and after the application of Schreckstoff, leaving a total of 22 trials for analysis. The nodes were then initialized with initial opinions drawn from the complex environment distribution and and were changed according to the opinion dynamics discussed in the main text.

Figure S3 A shows the distribution of the networks' normalized average degree before and after the treatment, indicating a clear change in favour of more densely connected structures. Panel B shows the probability of consensus, which is in agreement with our findings from Watts-Strogatz graphs, in that networks with higher normalized degree are more likely to reach consensus. Finally, panel C shows that the 'before' networks, although less likely to reach consensus show higher collective accuracy, indicting that they benefit from stalemate filtering.

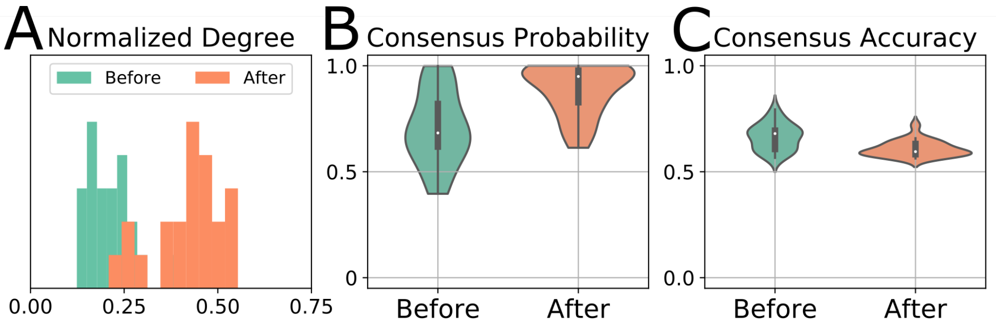

Figure S3. **The effect of stalemate filtering in biological networks** (A): normalized degree before (green) and after (orange) the application of Schreckstoff. The shift of the distribution mirrors the increase in cohesion. (B) The probability of reaching consensus increases in more densely connected networks (C) The consensus accuracy decreases in more densely connected networks. All three plots were generated using networks of 40 individuals. The edges were inferred from position data, considering two individuals connected whenever they were less than 16cm apart. To ensure comparability we restricted the analysis to those 22 networks that were connected both before and after the application of Schreckstoff.

Overall we could confirm that networks with lower normalized degree were more likely to end up in a stalemate but also showed the highest collective accuracy at consensus. This might indicate that an increase in cohesion as a response to external threat, might not only serve physical protection but also facilitate fast consensus decisions in situations where indecision might be fatal.

S5 Stalemate filtering can further improve optimal voting strategies

Previous work [6] has shown that if groups are operating in a complex environment, there is an optimal voting strategy (i.e an optimal value of p) which maximizes collective accuracy in a majority voting process. We compared this optimal strategy with stalemate filtering and found that groups using the optimal voting strategy can in fact outperform groups using stalemate filtering. However, if the optimal voting strategy is combined with stalemate filtering, groups perform better than when using any of these strategies alone. The results of this comparison are summarized in figure S4.

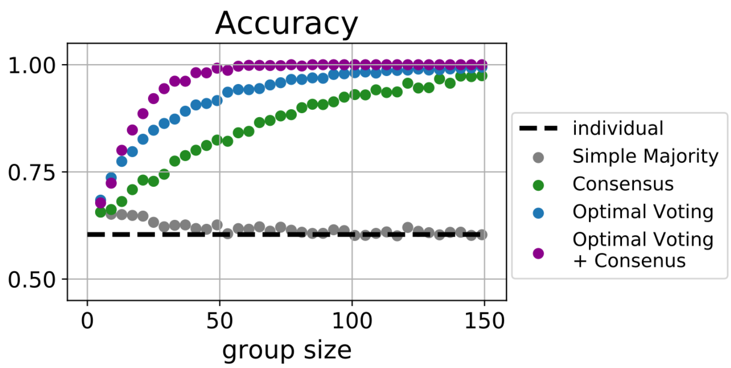

Figure S4. **Comparison of stalemate filtering with other voting strategies:** The different line colors show the results of different voting strategies. Grey: majority voting without interaction, $p = 0.6$. Green: Consensus decision after interaction $p = 0.6$. Blue: Majority voting using optimal value of voting strategy $P = p_{opt}$ calculated according to [6]. Purple: Consensus decision with initial opinions sampled using the optimal voting strategy p_{opt}. For all scenarios we used $r_I = 0.6$ and $r_C = 0.61$.

S6 The reward structure modulates the optimal collective decision-making process

We have found that the probability of stalemates, and the collective accuracy when consensus is reached, can be adjusted by changing the size of the group, the number of neighbors that an individual is connected to, and how clustered the network and the distribution of information are. Many of these properties may plausibly be tuned either through evolution or learning. However, the optimal configuration of parameters will depend on the relative costs and benefits of stalemates, correct decisions,

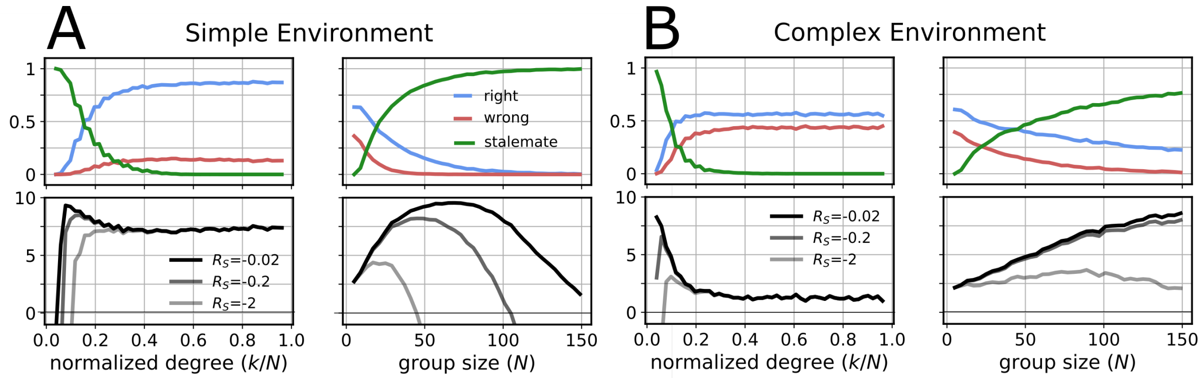

Figure S5. **Relative reward as a function of group size N and the normalized degree k/N) for simple (A) and complex (B) environments.** As before the default values are $N = 51$, $\beta = 0.2$ and $k = 5$ ($k/N \approx 0.098$). For the simple environment, we set $r = 0.58$, and in the complex environment we set $r_I = 0.6$, $r_C = 0.55$, and $p = 0.6$, such that in both environments, a solitary individual makes a correct decision with probability 0.58. The top row shows the probability of a single decision trial ending in consensus on the correct option (blue), consensus on the incorrect option (red) or a stalemate (green).
The bottom row shows a group's average received reward $\frac{1}{T}\sum_{t=1}^{T} R(t)$ where T is the total number of trials, and $R(t)$ is calculated according to equation 1. In all cases the reward for a consensus on the correct option is $+1$ and the punishment for a consensus on the incorrect option is -1. The punishment for each intermediate stalemate is varied between -0.02 (cost of stalemate is negligible) and -2 (cost of stalemate is higher than cost of a wrong decision). We find that for each environment, parameter type and stalemate cost, there is an optimal non-zero stalemate probability. Note that the average received reward can also become negative (e.g for very low normalized degree or very large group sizes in panel A). Since we can assume that parameters leading to negative reward will not be selected for, we we can ignore the exact values of negative reward and therefore do not extend the y-axis below 0.

and incorrect decisions. In the main text, we have focused on decision accuracy when consensus is reached, implicitly assuming that stalemates are relatively cost-free, compared to incorrect decisions. Most models of collective decision making, by contrast, do not allow for stalemates, therefore assuming that stalemates are highly costly. The true cost of stalemates will be context dependent. For some contexts, such as a group of animals hiding in a shelter, the cost of stalemates will be low relative to the potential cost of leaving the shelter (and potentially being eaten by a predator). For other contexts, stalemates may be highly costly, such as when a predator is attacking a group and fleeing in almost any direction may be better than remaining in place due to indecision.

Figure S5 illustrates the effect of two structural parameters (group size and normalized degree) on a group's relative reward, in both simple and complex environments. As a consequence of the results in the section *Detecting and Breaking Stalemates* we view a stalemate as a temporal delay in a decision process, which should ultimately be resolved via local or global redraws. For simplicity we here only consider global redraws, which correspond to a complete, memoryless repeat of a given decision trial. Consequently the total reward for a given decision trial $R(t)$ is composed of two parts:

$$R(t) = R_d + n_S C_S \tag{1}$$

where R_d is the reward or punishment for the final decision ($+1$ for a consensus on the correct option and -1 for a consensus on the incorrect option), C_S is the cost of a group getting stuck in a stalemate and n_S is the number of global redraws needed to resolve the stalemate and reach consensus.

Notably Figure S5 shows that for a given stalemate cost in all cases the optimal stalemate probability is non-zero, indicating that more often than not higher accuracy might be worth the price of a more difficult decision process.

References

1. Wickelgren, W. A. Speed-accuracy tradeoff and information processing dynamics. *Acta Psychol.* **41**, 67–85 (1977).

2. Franks, N. R., Dornhaus, A., Fitzsimmons, J. P. & Stevens, M. Speed versus accuracy in collective decision making. *Proc. Royal Soc. B* **270**, 2457–2463 (2003).

3. Barabási, A.-L. & Albert, R. Emergence of scaling in random networks. *science* **286**, 509–512 (1999).

4. Sosna, M. M. *et al.* Individual and collective encoding of risk in animal groups. *Proc. Natl. Acad. Sci.* **116**, 20556–20561 (2019).

5. Barthélemy, M. Spatial networks. *Phys. Reports* **499**, 1–101 (2011).

6. Kao, A. B., Miller, N., Torney, C., Hartnett, A. & Couzin, I. D. Collective learning and optimal consensus decisions in social animal groups. *PLOS Comput. Biol.* **10**, e1003762 (2014).
